# A randomized Phase III clinical trial to assess the efficacy of a bovine-human reassortant pentavalent rotavirus vaccine in Indian infants

**DOI:** 10.1016/j.vaccine.2017.09.014

**Published:** 2017-10-27

**Authors:** Prasad S. Kulkarni, Sajjad Desai, Tushar Tewari, Anand Kawade, Nidhi Goyal, Bishan Swarup Garg, Dinesh Kumar, Suman Kanungo, Veena Kamat, Gagandeep Kang, Ashish Bavdekar, Sudhir Babji, Sanjay Juvekar, Byomkesh Manna, Shanta Dutta, Rama Angurana, Deepika Dewan, Abhijeet Dharmadhikari, Jagdish K. Zade, Rajeev M. Dhere, Alan Fix, Maureen Power, Vidyasagar Uprety, Varsha Parulekar, Iksung Cho, Temsunaro R. Chandola, Vikash K. Kedia, Abhishek Raut, Jorge Flores, SII BRV-PV author group, Hanif Shaikh, Lalit Gupta, Rakesh Patil, Mohd. Aslam, Alok Arya, Farhana Rafiqi, Subodh S. Gupta, Chetna H. Maliye, P.V. Bahulekar, Kiran Bala, Tajali Nazir Shora, Shahid Hussain, Mihir Kumar Bhattacharya, Ashis K. Mukhopadhyay, Dilip Kumar Pal, Jayanta Saha, Ranjitha S. Shetty, Muralidhar M. Kulkarni, Chythra V. Raj

**Affiliations:** c Shirdi Saibaba Rural Hospital, Vadu, India; d Center for Health Research & Development, Society for Applied Studies, New Delhi, India; e Mahatma Gandhi Institute of Medical Sciences, Sewagram, India; f Government Medical College, Jammu, India; g National Institute of Cholera & Enteric Diseases, Kolkata, India; l Dr. BC Roy Post Graduate Institute of Paediatric Sciences, Kolkata, India; h Kasturba Medical College, Manipal, India; a Serum Institute of India Pvt Ltd, Pune, India; b PATH, Delhi, India; c Shirdi Saibaba Rural Hospital, Vadu, India; d Center for Health Research & Development, Society for Applied Studies, New Delhi, India; e Mahatma Gandhi Institute of Medical Sciences, Sewagram, India; f Government Medical College, Jammu, India; g National Institute of Cholera & Enteric Diseases, Kolkata, India; h Kasturba Medical College, Manipal, India; i Christian Medical College, Vellore, India; j PATH, Washington D.C., United States; k DiagnoSearch Pvt Ltd, Mumbai, India

**Keywords:** Rotavirus gastroenteritis, Infants, Vaccine, Efficacy, Safety

## Abstract

Rotavirus is the most common cause of moderate-to-severe infant diarrhoea in developing countries, resulting in enormous morbidity, mortality, and economic burden. A bovine-human reassortant pentavalent rotavirus vaccine (BRV-PV) targeting the globally most common strains was developed in India and tested in a randomized, double-blind, placebo-controlled end-point driven Phase III efficacy clinical trial implemented at six sites across India. Infants 6 to 8 weeks of age were randomized (1:1) to receive three oral doses of BRV-PV or placebo at 6, 10, and 14 weeks of age along with routine vaccines. Home visit surveillance was conducted to detect severe rotavirus gastroenteritis (SRVGE) and safety outcomes until the children reached two years of age. A total of 3749 infants received BRV-PV while 3751 received placebo. At the time of the primary end-point (when the minimum number of cases needed for analysis were accrued) the vaccine efficacy against SRVGE was 36% (95% CI 11.7, 53.6, p = 0.0067) in the per protocol (PP) analysis, and 41.9% (95% CI 21.1, 57.3, p = 0.0005) in the intent to treat (ITT) analysis. Vaccine efficacy over the entire follow-up period (until children reached two years of age) was 39.5% (95% CI 26.7, 50, p < 0.0001) in the PP analysis and 38.8% (95% CI, 26.4, 49, p < 0.0001) in the ITT analysis. Vaccine efficacy against the very severe rotavirus cases (VSRVGE, Vesikari score ≥ 16) was 60.5% (95% CI 17.7, 81, p = 0.0131) at the time of the primary analysis and 54.7% (95% CI 29.7, 70.8, p = 0.0004) for the complete follow-period in the PP population. The incidence of solicited, unsolicited, and serious adverse events were similar in both the vaccine and placebo groups. Likewise, the number of intussusceptions and deaths were similar between both groups. Thus, BRV-PV is an effective, well tolerated and safe vaccine in Indian infants. (Trial registration: Clinical Trials.Gov [NCT 02133690] and Clinical Trial Registry of India [CTRI/2013/05/003667]).

## Highlights

• Pentavalent reassortant rotavirus vaccine was tested for efficacy in infants.
• The vaccine (BRV-PV) showed excellent tolerability and a good safety profile.
• Primary analysis efficacy was 36% against SRVGE and up to 60.5% against VSRVGE.
• The efficacy through 2 years of age was 39.5% (SRVGE) and 54.7% (VSRVGE).
• The intent to treat analyses confirmed all the per protocol analyses.

## 1 Introduction

As per an estimate for 2013, 37% of the 578,000 diarrhoeal deaths worldwide in children less than 5 years of age were caused by rotavirus that year, leading to 215,000 deaths in this age group; most of which were in Asia and Africa, with around 22% in India [1]. In India alone, it is estimated that 11.37 million episodes of rotavirus gastroenteritis occur every year, requiring 3.27 million outpatient visits and 872,000 inpatient admissions [2].

Currently two rotavirus vaccines (Rotarix and RotaTeq) are licensed internationally and are World Health Organization (WHO) prequalified [3], [4]. A third vaccine (ROTAVAC) was recently licensed in India [5]. Despite this, there remains an overwhelming need for cost-effective and safe rotavirus vaccines for the countries that need them the most.

Using indigenous manufacturing technology at Serum Institute of India Pvt Ltd (SIIPL), a pentavalent rotavirus vaccine (BRV-PV) was developed from five Bovine (UK) X Human Rotavirus Reassortant strains (serotypes G1, G2, G3, G4, and G9) received from the US National Institutes of Health (NIH). Initial studies on a quadrivalent formulation of the G1, G2, G3, and G4 reassortants conducted in the US and Finland demonstrated that they are safe, well tolerated, immunogenic, and efficacious [6], [7], [8]. Three clinical studies (one Phase I and two Phase II) were conducted with the BRV-PV in India. The vaccine was found safe and immunogenic in these studies, and the immunogenicity results were similar to those reported for the licensed vaccines in India [9].

We conducted a large Phase III study to assess the clinical efficacy of the vaccine in preventing severe rotavirus gastroenteritis (SRVGE) and expand on the safety observations made in the earlier trials. This paper gives results from the complete analysis of the study.

## 2 Material and methods

The trial was a double-blind, randomized, placebo-controlled, endpoint-driven study to assess the efficacy and safety of the BRV-PV in healthy Indian Infants. Subjects were followed from the time of vaccination until 2 years of age for efficacy and safety outcomes. During this period, there were 8 visits by subjects to the study clinics, in addition to active surveillance consisting of weekly home visits by the field workers (FWs) to detect, characterize and collect stool specimens from all occurring episodes of gastroenteritis (GE). The study was initiated in May 2014 and conducted at clinical sites across six cities in India (Pune, Kolkata, Sewagram, Delhi, Manipal, and Jammu). The results presented in this paper represent all the safety data gathered for the vaccine, as well as efficacy data collected when the primary efficacy endpoint was met and efficacy data for the entire follow-up, i.e., through 2 years of age. The subjects were randomized to receive three oral doses of BRV-PV or placebo at 6, 10, and 14 weeks of age along with the routine DTP-HB-Hib and oral polio vaccines. There was no restriction on breastfeeding around the time of vaccination. The parents were provided with mobile phones to ensure prompt contact with the study team in case the infants had gastroenteritis or any other illness. The parents were also given a diarrhoea diary card (DDC) to capture signs and symptoms of gastroenteritis including diarrhoea, vomiting, and fever, instructed in its completion, and provided with a digital thermometer to monitor body temperature. Diarrhoea was defined as 3 or more watery or looser than normal stools within 24 h. Parents were instructed to notify the study team on the onset of diarrhoea, complete the DDC and collect a stool sample in containers provided to them. The FWs visited the families of all subjects with GE and reviewed the DDCs; GE cases were evaluated for dehydration based on the WHO criteria [10] by trained FWs or study physicians. Sick subjects received prompt medical care by study physicians and by the referral hospitals. Visits continued on alternate days until the GE episode resolved to ensure the wellbeing of the child as well as appropriate data capture on the DDC. A GE episode was considered resolved when the child was free from diarrhoea for at least 5 consecutive days.

The information from the DDC was used to grade the GE episodes as per the Vesikari Scoring System [11]. An episode of GE with a score of ≥11 was classified as ‘severe’ and constituted an endpoint for the primary analysis. Episodes with a ≥16 score were classified as ‘very severe,’ 7–10 as ‘moderate,’ and less than 7 as ‘mild’ GE.

Stool samples were tested at the Welcome Trust Research Laboratory, Christian Medical College, Vellore for rotavirus by a validated ELISA assay [12]. Rotaviruses detected in stool samples were further characterised for genotypes by reverse transcriptase polymerase chain reaction (RT-PCR). If an ELISA positive sample was found un-typable for both VP7 and VP4 genes, the sample was tested for VP6 gene by PCR. If the VP6 was negative in RT-PCR, the result of the ELISA was considered false positive. If the VP6 was positive, nucleotide sequencing was attempted in order to complete the genotyping. All of the tests were conducted under Good Laboratory Practice (GLP) standards.

A case of SRVGE constituted a clinical endpoint for the primary analysis of the study. For children experiencing more than one episode of SRVGE, only the first episode was counted towards the primary endpoint.

This was an event-driven study in which the primary analysis was assessment of efficacy against SRVGE occurring after 14 days from the last dose among subjects who received all 3 doses. This primary analysis was to take place when at least 122 cases of SRVGE were accrued or when all the subjects reached 2 years of age, whichever was earlier. This report provides vaccine efficacy data collected up to 11 October 2015, when 155 cases had accrued (primary analysis), as well as a full 2-years of efficacy data (complete analysis).

### 2.1 Safety assessment

The enrolled subjects were followed for safety outcomes through 2 years of age, including severe unsolicited adverse events (AEs), serious adverse events (SAEs), deaths, and the occurrence of intussusception. Close monitoring for post-vaccination safety was conducted in a sub-cohort of 1009 infants (reactogenicity cohort). Parents of children in this reactogenicity cohort were given a post-immunization diary card (PIDC) to record solicited reactions (diarrhoea, fever, vomiting, decreased appetite, decreased activity level, and irritability) daily for 7 days post vaccination. To ensure compliance, FWs made home visits on Day 3 (+2) after each vaccination, when the PIDC was reviewed and the parents were questioned about unsolicited events. An additional home visit was made on Day 7 (−1 to +2) to review and collect the PIDC. Infants in the reactogenicity cohort were closely monitored until 28 days after the third investigational product dose and all unsolicited AEs of any severity grade were documented.

A protocol safety review team (PSRT) comprised by physicians from SIIPL, PATH, and DiagnoSearch Pvt Ltd (the contract research organization supporting the study) continuously monitored the safety findings in the study. An independent data safety monitoring board (DSMB) comprised of independent physicians and biostatistician provided additional oversight on the study subjects’ safety.

Surveillance for intussusception was maintained throughout the study. Study physicians were trained to identify warning signs and ensure prompt evaluation and treatment as indicated. All potential cases were reviewed by an independent intussusception adjudication committee (IAC) that included a paediatric surgeon, a paediatrician, and a radiologist. Cases determined by the IAC as meeting criteria for Level 1 of diagnostic certainty according to the Brighton Collaboration definition of intussusception [13] were reported as intussusception.

### 2.2 Study vaccines

The BRV-PV is a live attenuated pentavalent rotavirus vaccine containing five single gene substitution reassortants between human strains G1, G2, G3, G4, and G9 and the bovine UK strain. The viruses were grown in VERO cells at a titre of ≥10^5.6^ FFU/serotype/dose. The vaccine was formulated as a lyophilized powder containing5% sucrose and glycine as stabilizer. The placebo was a lyophilized powder containing all the vaccine constituents except the viruses. Citrated sodium bicarbonate containing 25.6 g of sodium bicarbonate and 9.6 g citric acid per litre available in a separate vial was used as diluent for both vaccine and placebo. The vaccine and placebo were visually undistinguishable. The BRV-PV is stable at 25 °Cfor 30 months and at 40 °Cfor 18 months. Since this was the pivotal trial to support licensure, the study vaccines were transported and stored at 2–8 °C out of caution. All the study vaccine containers looked identical with unique vial numbers printed on them to maintain blinding.

Four batches of BRV-PV were used during the study; 145O2001, 145O2002, 145O2003, and 145E40010Z. The batches were consistent in virus titres of all five serotypes as follows: Batch 145E0010Z-titre range 6.172–6.241 Log_10_FFU/vial, Batch 145O2001-titre range 5.996–6.051 Log_10_FFU/vial. Batch 145O2002-titre range - 5.934–6.027 Log_10_FFU/vial, Batch 145O2003-titre range 5.971–6.033 Log_10_FFU/vial.

Eligible subjects were randomly assigned to receive vaccine or placebo in a 1:1 ratio using a computer-generated allocation schedule. Randomization was stratified by sites and a block size of 6 was used to ensure 1:1 balance within each site. The assignments were provided to the sites by a validated interactive web response system (IWRS). After obtaining consent, a unique subject identification code (Subject ID) was allocated to each subject through IWRS and a corresponding study vaccine kit was used for that subject.

Each kit contained one vial of lyophilized vaccine/placebo, one vial of diluent, one vial adapter and one 5 mL syringe for vaccine reconstitution. For reconstitution, the adapter was fixed to the syringe and the syringe/adapter was connected to the diluent vial. By pulling the plunger back, diluent was aspirated into the syringe. The assembly was removed from diluent vial and was connected to the vaccine/placebo vial. The entire content of the syringe was injected into the vial. By pulling the plunger back, 2.5 mL of reconstituted solution was aspirated from vial into the syringe. Then the syringe was removed from the adapter and was ready for administration. The vaccine was used within one hour of reconstitution though the reconstituted vaccine is stable for 24 h at 2–8 °C and at room temperature and for 3 h at 40 °C. If the subject drooled or spit the product, the dose was not repeated. If a subject vomited within 5 min after study vaccination, or if the kit was damaged, a backup kit was allocated to the subject through the IWRS system.

### 2.3 Study participants

The study was conducted at six urban/semi-urban/rural study sites across India that represent different climatic, geographical, and sociocultural environments. The subjects were 6- to 8-week-old healthy infants whose parents gave written informed consent and who were residents of the study area. Infants with ongoing diarrhoea or vomiting, fever, or any acute disease were temporarily excluded. Other key exclusion criteria were: significant malnutrition (weight-for-height Z score ≤ −3 SD, median) [14]; any systemic disorder; congenital abdominal disorder; history of persistent diarrhoea, intussusception, or abdominal surgery; suspected immune compromised status; allergy to any components of the study vaccines; or known major congenital defect. If a study subject suffered from an immediate hypersensitivity reaction following vaccination, significant illness, underwent major surgery, had a protocol violation, or received licensed rotavirus vaccine prior to receipt of all study doses, the infant did not receive subsequent vaccination, though the safety follow up continued.

### 2.4 Immunological assays

A sub-cohort of 219 randomly selected infants from among those enrolled early in the study (immunogenicity cohort) was examined for IgA seroresponses against rotavirus and neutralizing antibody responses against the three poliovirus vaccine serotypes. Serum samples from these infants were obtained just before vaccination and 4 weeks after the third vaccine dose. A seroresponse for rotavirus was defined as ≥3-fold increase in IgA titre between post-vaccination and pre-vaccination sera. The IgA was analysed at the Welcome Trust Research Laboratory, Christian Medical College laboratory in Vellore by a conventional ELISA assay using rotavirus-infected cell culture lysate [15]. The polio neutralization assays were conducted at the Enterovirus Research Centre in Mumbai following WHO-recommended procedures [16].

### 2.5 Statistical analysis

The trial was event-driven with the primary efficacy analysis to be triggered by the accrual of 122 SRVGE cases (primary endpoint). Under the assumptions of 50% true vaccine efficacy (95% CI lower bound 10%), attack rate of SRVGE of 2.5%, and drop-out rate of 15%, 7500 infants were required to achieve statistical power of 90%. The point estimate of the vaccine efficacy (VE) for SRVGE, i.e., (1-Hazard Ratio of the vaccine group, relative to the placebo group) × 100%, and the 95% CI were calculated using the Cox Proportionate Hazards model. The World Wald Test based on the Cox model was calculated at a 1-sided level of 0.025 (this test rejects H₀ if and only if the 2-sided 95% CI for VE lies above 10%). A successful outcome for the vaccine was defined as lower bound of two sided 95% CI more than 10%, thus demonstrating efficacy of the vaccine.

Two efficacy populations were analysed. The Per Protocol (PP) population included all subjects who received all three doses of study vaccine/placebo as per the randomization by 32 weeks of age and were free from any major protocol deviation. The RVGE endpoints in this population were counted from 14 days after the third vaccination. The Intent-to-Treat (ITT) population included all randomized subjects receiving at least one vaccine/placebo dose (including those receiving an incorrect product). Primary endpoints in this population were counted from the time of the first vaccination. The safety population included all the enrolled subjects.

The PP population was the dataset for the primary efficacy analysis. Secondary efficacy endpoints were tested on both the PP population and the ITT population and included: VE against RVGE of any severity, VE against SRVGE in the first year of life, VE against SRVGE over the entire follow-up period, VE against SRVGE associated with rotavirus serotypes included in the vaccine, VE against SRVGE with hospitalization (≥24 h), VE against SRVGE with dehydration (defined as per WHO criteria [10]), and vaccine impact on the incidence of SRVGE. An additional post hoc analysis of vaccine efficacy against very severe RVGE (VSRVGE) was conducted on the subset of cases with a Vesikari score ≥16.

Safety endpoints were analysed for all infants enrolled (safety population). They included: immediate AEs within 30 min of each vaccination; severe unsolicited AEs; SAEs; GE events; death; and intussusceptions. In addition, solicited AEs within 7 days of each vaccination and unsolicited AEs of any grade until 28 days after last vaccination were examined in the reactogenicity cohort of infants. Exact p values were computed for solicited AEs and selected unsolicited AEs. All safety endpoints were presented in numbers and percentages. Gender was presented in percentages while age, weight, and length were presented with mean, median, and standard deviation. All analyses were performed using SAS® version 9.2.

For the immunological assays, the number and percentage (%) of subjects with seroresponses to rotavirus and polio serotypes 1, 2, and 3 are presented by study arm along with two-sided exact 95% CI based on the Clopper-Pearson method. Seroconversion for rotavirus vaccine was defined as percentage of subjects with ≥3-fold increase in rotavirus IgA titres at Day 28 (±7 days) post dose 3 with respect to baseline values. Seroconversion for polio antibodies was defined as (a) for participants seronegative at the baseline time point, neutralizing antibody titres >1:8; and (b) for participants seropositive at the baseline time point, neutralizing antibody titres that are ≥4-fold higher than the expected titre of maternal antibodies based on the pre-vaccination titre declining with a half-life of 28 days. The comparison of the study arms was carried out by constructing the rate difference between the study arms and the corresponding two-sided 95% CI using the Wilson Score method.

### 2.6 Ethical considerations

The study was performed in accordance with the International Conference on Harmonisation (ICH) Guidelines for Good Clinical Practice (GCP) E6 (R1), GCP guidelines by the Central Drug Standard Control Organization Government of India, Schedule Y, Amended 2005 under Drugs and Cosmetics Act and Rules, and the Declaration of Helsinki, 2013. All subjects were enrolled only after written consent of their parents. The process of consent was recorded audio-visually as required by prevalent regulations in India. The study was approved by the Drugs Controller General of India as well as by the respective Institutional Ethics Committees of all the participating sites and the Western Institutional Review Board, Puyallup, Washington, USA. All subjects were given prompt medical care for any health issues.

## 3 Results

**Fig. 1:**
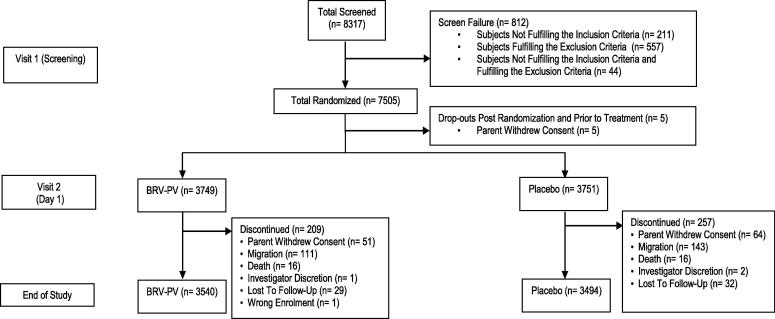
Study flowchart.

Of the 7505 subjects randomized, 5 did not receive any doses (parents withdrew consent before vaccination). A total of 7500 infants were enrolled in the study, of which 3749 received the BRV-PV and 3751 received placebo (Fig. 1). Of the 7500 infants, 252 received only the first dose, 88 received only two doses, and 7161 (95.4%) went on to receive all three doses (Table 1). A total of 3781 participants (50.4%) were male. The mean age at baseline was 48 days in both groups. No differences in weight at birth, or weight or length at enrolment, were observed between the vaccine and placebo groups (Table 2). At the time of the primary analysis, 7237 subjects were part of the study. Of these 7025 subjects were available for PP analysis. (Table 1). A total of 7034 subjects completed the study and 466 subjects discontinued the study before completion due to reasons like withdrawal of consent, migration, death, investigator discretion and lost to follow up (Fig. 1). A total of 7035 subjects were part of PP population for the final efficacy analysis (Table 1).

**Table 1:** Summary of subject disposition.

Subject disposition	BRV-PV	Placebo	Total
	(N = 3750)	(N = 3755)	(N = 7505)
Number of subjects randomized	3750 (100.00%)	3755 (100.00%)	7505 (100.00%)
Number of subjects received at least one dose	3749 (99.97%)	3751 (99.89%)	7500 (99.93%)
Number of subjects who have received all three doses	3595 (95.87%)	3566 (94.94%)	7161 (95.40%)
Intent to treat (ITT) population	3749 (99.97%)	3751 (99.89%)	7500 (99.93%)
Number of subjects in the study at time of Primary Analysis	3644 (97.2%)	3593 (95.78%)	7237 (96.5%)
Per protocol population at the time of primary analysis	3527 (94.05%)	3498 (93.16%)	7025 (93.60%)
Subjects who have completed two year of age follow-up	3540 (94.4%)	3494 (93.04%)	7034 (93.7%)
Per protocol population at the time of final analysis	3533 (94.2%)	3502 (93.2%)	7035 (93.73%)
Safety population	3749 (99.97%)	3751 (99.89%)	7500 (99.93%)
Reactogenicity cohort	502 (13.39%)	507 (13.50%)	1009 (13.44%)
Immunogenicity cohort	116 (3.09%)	103 (2.74%)	219 (2.92%)

**Table 2:** Demographics and baseline characteristics (% or mean and SD).

Characteristic	BRV-PV	Placebo	Total
	(N = 3749)	(N = 3751)	(N = 7500)
Age (days) Dose 1	48.3 (4.05)	48.2 (4.11)	48.2 (4.08)
Age (days) Dose 2	84.5 (19.86)	84.9 (22.19)	84.7 (21.06)
Age (days) Dose 3	120.8 (26.63)	120.9 (27.99)	120.9 (27.32)
Age (days) at the time of Primary analysis	373.2 (77.89)	373.3 (77.92)	373.2 (77.90)
Male n (%)	1858 (49.6%)	1923 (51.3%)	3781 (50.4%)
Weight at birth (kg)	2.8 (0.41)	2.8 (0.41)	2.8 (0.41)
Weight at baseline (kg)	4.3 (0.58)	4.3 (0.58)	4.3 (0.58)
Length at baseline (cm)	54.7 (2.25)	54.7 (2.23)	54.7 (2.24)

### 3.1 Primary efficacy analysis

For the primary endpoint, the PP population analysis of efficacy included 61 cases of SRVGE reported in the vaccine group and 94 cases reported in the placebo group for a vaccine efficacy of 36% (95% CI 11.7, 53.6, p: 0.0067) (Table 3). For the corresponding ITT analysis, including all 7500 infants receiving at least one dose, 65 cases of SRVGE were reported in the vaccine group and 110 cases in the placebo group for a vaccine efficacy of 41.9% (95% CI 21.1, 57.3, p: 0.0005). For VSRVGE (Vesikari score ≥ 16) in the PP population, 10 and 25 cases were reported in the vaccine and placebo groups, respectively, for an efficacy of 60.5% (95% CI 17.7, 81.0, p: 0.01); the corresponding VE for VSRVGE in the ITT population was 61.3% (95% CI 22.2, 80.7, p: 0.007) (Table 3).

**Table 3:** Summary of vaccine efficacy at the time of primary analysis.

	Per protocol analysis					Intent to treat analysis				
	BRV-PV	Placebo	Vaccine efficacy		p-value	BRV-PV	Placebo	Vaccine efficacy		p-value
	N = 3527	N = 3498	%	95% CI		N = 3749	N = 3751	%	95% CI	
SRVGE	61	94	36	11.7, 53.6	0.0067	65	110	41.9	21.1, 57.3	0.0005
Very severe RVGE	10	25	60.5	17.7, 81.0	0.0131	11	28	61.3	22.2, 80.7	0.0077
RVGE of any severity	144	197	28.3	11.1, 42.2	0.0024	162	245	35.5	21.4, 47.1	<0.0001
SRVGE in first year of life	52	78	34.1	6.3, 53.6	0.02	56	93	40.6	17.3, 57.4	0.002
SRVGE against vaccine serotypes	61	92	34.6	9.6, 52.7	0.0102	65	108	40.8	19.5, 56.5	0.0008
SRVGE requiring hospitalization	35	53	34.8	0.0, 57.4	0.0498	37	63	42.2	13.2, 61.5	0.0082
Severe GE of any etiology	364	379	5	−9.7, 17.8	0.4825	472	509	7.5	−4.9, 18.5	0.2221

**Fig. 2:**
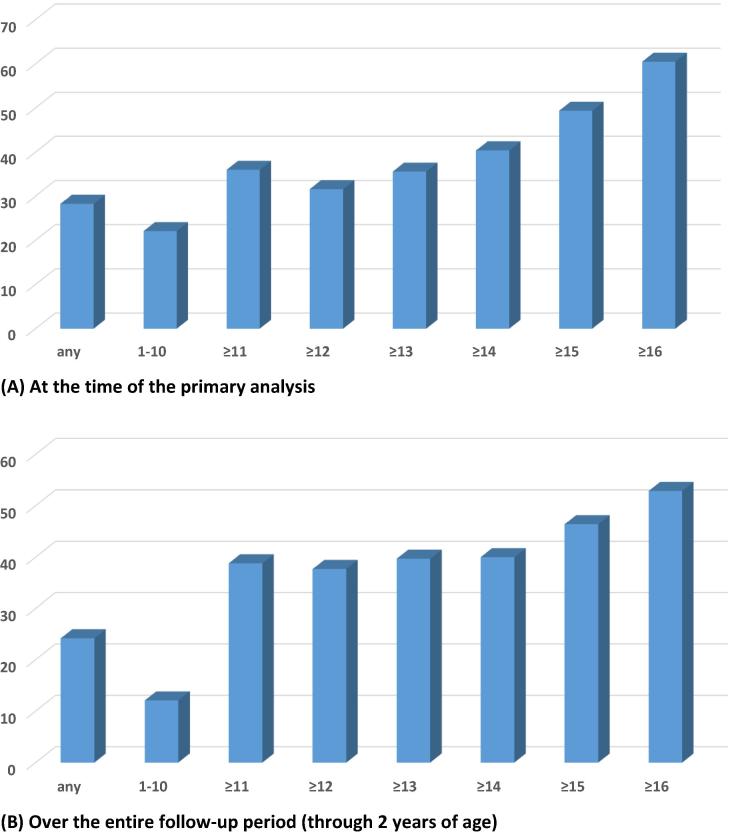
Relationship between Vesikari severity score and corresponding vaccine efficacy.

By the time of the primary analysis, a total of 144cases of RVGE of any severity had been reported in the vaccine group and 197 in the placebo group for an efficacy of 28.3% (95% CI 11.1, 42.2, p: 0.0024). The corresponding VE in the ITT population was 35.5% (95% CI 21.4, 47.1, p: <0.0001) (Table 3). The levels of efficacy improved as the severity of RVGE increased (Fig. 2 and Supplemental Table 1).

By the time of the primary analysis 35 cases of SRVGE had been hospitalized in the vaccine group and 53 cases in the placebo group (efficacy 34.8%, 95% CI 0.0, 57.4, p = 0.05) For the ITT population, the efficacy against SRVGE with hospitalization was 42.2% (95% CI 13.2, 61.5, p = 0.008) (Table 3).

### 3.2 2-year efficacy analysis

Over the entire follow-up period (through 2 years of age) the efficacy analysis included 171 cases of SRVGE reported in the vaccine group and 275 cases reported in the placebo group for a vaccine efficacy of 39.5% (95% CI 26.7, 50, p < 0.0001) (Table 4). For the corresponding ITT analysis, 186 cases were reported in the vaccine group and 296 in the placebo group for a vaccine efficacy of 38.8% (95% CI 26.4, 49.0, p < 0.0001) (Table 4). For VSRVGE (Vesikari score ≥ 16) in the PP population, 29 and 63 cases were reported in the vaccine and placebo groups, respectively, for an efficacy of 54.7% (95% CI 29.7, 70.8 p = 0.0004); the corresponding VE for VSRVGE in the ITT population was 52.9% (95% CI 28.2, 69.1, p = 0.0005) (Table 4).

**Table 4:** Summary of vaccine efficacy at the time of final analysis.

	Per protocol analysis					Intent to treat analysis				
	BRV-PV	Placebo	Vaccine efficacy		p-value	BRV-PV	Placebo	Vaccine efficacy		p-value
	N = 3533	N = 3502	%	95% CI		N = 3749	N = 3751	%	95% CI	
SRVGE	171	275	39.5	26.7, 50.0	<0.0001	186	296	38.8	26.4, 49.0	<0.0001
Very severe RVGE	29	63	54.7	29.7, 70.8	0.0004	32	67	52.9	28.2, 69.1	0.0005
RVGE of any severity	492	614	22.6	12.9, 31.3	<0.0001	531	675	24.2	15.1, 32.4	<0.0001
SRVGE in first year of life	85	125	32.9	11.6, 49.1	0.0045	90	140	36.6	17.4, 51.3	0.0008
SRVGE against vaccine serotypes	170	271	38.9	26.0, 49.6	<0.0001	185	292	38.2	25.7, 48.6	<0.0001
SRVGE requiring hospitalization	95	140	33.4	13.6, 48.7	0.0022	101	153	35.1	16.6, 49.5	0.0007
Severe GE of any etiology	804	832	4.6	−5.1, 13.4	0.3417	934	979	5.1	−3.9, 13.2	0.2578

For the 2-year analysis, 492 cases of RVGE of any severity were reported in the vaccine group and 614 in the placebo group for an efficacy of 22.6% (95% CI 12.9, 31.3, p < 0.0001) in the PP population. The corresponding VE in the ITT population was 24.2% (95% CI 15.1, 32.4, p < 0.0001) (Table 4).

Over the entire follow-up period the SRVGE cases with hospitalization were 95 in the vaccine group and 140 in the placebo group (VE 33.4% 95% CI, 13.6, 48.7, p = 0.002) in the PP population, and 101 cases in the vaccine group and 153 in the placebo group (VE 35%, 95% CI, 16.6, 49.5, p = 0.0007) in the ITT population (Table 4).

Overall, vaccination resulted in a drop in SRVGE incidence of 1.77 cases per 100 person-years from 4.56 in the placebo group to 2.79 in the vaccine group (ITT population) over the two years of follow-up.

The great majority of SRVGE cases (∼90%) over the entire period of follow-up were associated with the serotypes included in the vaccine, i.e., serotypes G1, G2, G3, G4, and G9 (range: 34–74 cases). Only a few cases ofG10 and G12 rotaviruses were identified. The efficacy analysis for SRVGE cases due to the G serotypes represented in the vaccine included 185 cases reported in the vaccine group and 292 cases reported in the placebo group, for an efficacy of 38.2% (95% CI 25.7, 48.6, p<0.0001) in the ITT population (Table 4 and Supplemental Table 2). The corresponding vaccine efficacy for primary analysis in the ITT population was 40.8% (95% CI 19.5, 56.5, p = 0.0008) (Table 3).

### 3.3 Safety results

There were two AEs occurring within 30 min of vaccination among vaccine recipients, one each of discoloration of oral mucosa observed after the second dose and decreased appetite following the first dose. The events were mild in severity and brief induration. Such events did not recur with subsequent doses. There were 19 and 12 episodes of vomiting within 5 min of receiving BRV-PV and placebo, respectively.

### 3.4 Reactogenicity cohort

Solicited AEs (including diarrhoea, fever, vomiting, decreased appetite, decreased activity level, and irritability) were recorded during the 7 days post each vaccination for subjects in the reactogenicity cohort (n = 1009). A total of 1347 solicited AEs in 399 subjects were observed in the vaccine group and 1281 events in 400 subjects in the placebo group. The proportions of subjects reporting solicited events of fever, decreased appetite, decreased activity, vomiting, and diarrhoea were similar between study arms (p = 0.386–1.0, Fisher’s exact test), however irritability was reported with higher frequency in the vaccine group (42.6%) in comparison to the placebo group (36.1%). Most of the cases of irritability were mild and there were no grade 3 cases of irritability in the study. The results are presented in Table 5 and Supplemental Table 3. The solicited reactions were generally mild, and of short duration except for 5 cases of severe fever, defined as a temperature of ≥39.5 – <40.5 °C (4 in the BRV and 1 in the placebo group). The incidence of solicited reactions was lower after the second and third dose as compared to the first (Supplemental Table 3). Unsolicited AEs were observed up to 28 days post-last dose in the reactogenicity cohort. A total of 361 subjects in the BRV-PV group and 321 subjects in the placebo group (p = 0.0002, Fisher’s exact test) reported a total of 1476 unsolicited events up to 28 days post-last dose. Lower respiratory tract infections occurred in greater number of subjects in BRV-PV group (n = 60) as compared to placebo group (n = 38) (p-0.019, Fisher’s exact test). These findings were not corroborated in the safety population which included all the randomized children (see below).

**Table 5:** Incidence of solicited adverse events all three doses combined – reactogenicity cohort.

Solicited AE	BRV-PV	Placebo	P value (Fisher’s Exact test)
	(N = 500)	(N = 509)	
	n%, E	n%, E	
Fever	341 (68.2%), 581	355 (69.7%), 593	0.634
Irritability	213 (42.6%), 347	184 (36.1%), 290	0.039
Decreased appetite	102 (20.4%), 144	102 (20.0%), 134	0.938
Decreased activity	94 (18.8%), 123	87 (17.1%), 109	0.512
Vomiting	85 (17.0%), 109	86 (16.9%), 101	1
Diarrhoea	42 (8.4%), 43	51 (10.0%), 54	0.386

n = number of events; E = Number of all reported events including multiple occurrences of same event children; % = Percentage is based on number of subjects in reactogenicity cohort for each group (N).

### 3.5 Safety population

Safety evaluations in the safety population, which included all randomized children, were only recorded for grade 3 or higher AEs. A total of 250 subjects (6.6%) in the placebo group and 222 subjects (5.9%) in the vaccine group reported 269 and 241 severe non-serious AEs, respectively, through the end of the study. The proportions of subjects with non-serious unsolicited events were similar in both groups (p-0.199, Fisher’s exact test).

A total of 3320 SAEs were reported in 2225 of the 7500 enrolled subjects (29.6%) by the time of final analysis; they were experienced by 1109 subjects in the BRV-PV group and 1116 subjects in the placebo group (Table 6). The proportion of subjects with SAEs between the study arms was similar (P = 0.879, Fisher's exact test). The type and incidence of unsolicited events and SAEs observed from the time of the first vaccination through the time of the primary analysis are typical of the background morbidity in the Indian infant population and included lower respiratory tract infection (8.38% of vaccine recipients, 8.42% in placebo recipients); gastroenteritis (8.24% of vaccine recipients, 9.09% of placebo recipients); pyrexia (2.77% of vaccine recipients, 2.69% of placebo recipients); bronchiolitis (2.32% of vaccine recipients, 2.08% of placebo recipients) and bronchopneumonia (2.00% of vaccine recipients, 2.21% of placebo recipients). None of the SAEs led to study discontinuation over this period. There were 16 deaths in the BRV-PV arm and 16 deaths in the placebo arm, and none were related to the study products. Causes of deaths were diverse and included infections such as meningitis, pneumonia, encephalitis, dengue, aspiration, acute leukaemia, septic shock, sudden infant death syndrome, and multi-organ failure following surgery for congenital heart defect (Supplemental Table 4). Thirteen cases of intussusception were diagnosed; 6 occurred in the BRV-PV arm and 7 in the placebo arm. None occurred within 28 days of receiving a dose of BRV-PV or placebo. None were related to study vaccination or led to discontinuations from the study.

**Table 6:** Overview of serious adverse events post dose 1 up to 2 years of age – safety population.

SAE details	BRV-PV	Placebo	Total	P value
	(N = 3749)^a^ n%, E	(N = 3751) n%, E	(N = 7500) n%, E	(Fisher’s Exact test)
At least one SAE	1109 (29.58%), 1677	1116 (29.75%), 1643	2225 (29.67%), 3320	0.879
Life-threatening	71 (1.89%), 75	61 (1.63%), 61	132 (1.76%), 136	0.382
Severe	682 (18.19%), 930	662 (17.65%), 906	1344 (17.92%), 1836	0.547
Moderate	342 (9.12%), 646	376 (10.02%), 648	718 (9.57%), 1294	0.195
Mild	14 (0.37%), 26	17 (0.45%), 28	31 (0.41%), 54	0.720
At least one related^b^ SAE	7 (0.19%), 7	5 (0.13%), 5	12 (0.15%), 12	0.581
At least one SAE resulting in death	16 (0.43%), 16	16 (0.43%), 16	32 (0.43%), 32	1.000
Intussusception	6 (0.16%), 6	7 (0.19%), 7	13 (0.17%), 13	1.000

Serious Adverse Events (SAEs) were any AEs that resulted in death, were life threatening, required inpatient hospitalization, resulted in prolongation of existing hospitalization, resulted in persistent or significant disability/incapacity, or were a congenital anomaly/birth defect.

a N = number of infants enrolled; n = number of infants with SAE (% over N), E = number of SAE episodes.

b Related: There is a reasonable causal relationship between study product administered and the AE.

IgA seroresponses were demonstrated in 39 of 116 (33.6%) vaccine recipients in the immunogenicity cohort, and 10 of 103 placebo recipients (9.7%) (Table 7). Seroconversions to poliovirus serotypes 1, 2, and 3 were observed in 96.6%, 96.6% and 92.2% of infants, respectively, in the vaccine group, and 97.1%, 100%, and 94.2%, respectively, in the placebo group (Table 8).

**Table 7:** IgA seroresponses to rotavirus.

	BRV-PV	Placebo
	(N = 116)	(N = 103)
Number of subjects for whom results are available	116	103
Number of Subjects with Rotavirus IgA Titers ≥20 IU/mL (Seropositive) at baseline	15	8
Number of subjects achieving a ≥3-fold rise at Day 28 (±7 Days) post-dose 3	39	10
Percentage of subjects achieving a ≥3-fold rise	33.6%	9.7%
Two-sided 95% CI for percentage: (LCL, UCL)	(25.1%, 43.0%)	(4.8%, 17.1%)

**Table 8:** Seroresponses to concomitantly administered oral polio vaccine.

	BRV-PV	Placebo	Group difference Placebo, BRV-PV
	(N = 116)	(N = 103)	
	n (%)	n (%)	
*Type 1*			
Percentage of subjects showing seroconversion	112 (96.6%)	100 (97.1%)	0.5%
Two-sided 95% CI for percentage (LCL, UCL)	91.4%, 99.1%	91.7%, 99.4%	−5.2%, 6%

*Type 2*			
Percentage of subjects showing seroconversion	112 (96.6%)	103 (100.0%)	3.4%
Two-sided 95% CI for percentage (LCL, UCL)	91.4%, 99.1%	96.5%, 100%	−0.7%, 8.5%

*Type 3*			
Percentage of subjects showing seroconversion	107 (92.20%)	97 (94.20%)	1.90%
Two-sided 95% CI for percentage (LCL, UCL)	85.8%, 96.4%	87.8%, 97.8%	−5.3%, 9%

## 4 Discussion

The study demonstrated efficacy of BRV-PV against SRVGE (36%), the primary study endpoint, as well as against RVGE of any severity (28.3%). These efficacy levels were maintained through the second year of life, with an overall efficacy of 39.5% against SRVGE and 22.6% against RVGE of any severity. All of the pre-established secondary efficacy endpoints confirmed these findings as well as the ITT analyses. The vaccine prevented 42.5% of hospitalizations due to SRVGE in the primary analysis, and 35.1% over the full 2-year follow-up (ITT analysis). This has great significance in countries like India where access to medical care is often difficult. Another significant finding was that BRV-PV prevented more than 50% of very severe rotavirus infections, which represent the highest risk of dehydration, hospitalization, and death. This was shown both, at the time of the primary analysis as well as at the completion of the follow-p through two years.

RotaTeq and Rotarix have shown high levels of efficacy in developed countries, ranging from 98% (95% CI 88–100) in the United States and Finland [17] to 85% (95% CI42-97) in Finland alone [18]. A quadrivalent vaccine containing four of the five strains present in the BRV-PV showed a high level of efficacy (90%) in a small study in Finland [8]. However, when tested in the developing world, the efficacy of RotaTeq and Rotarix has been shown to be diminished. The efficacy of RotaTeq has been as low as39.3% (95% CI 19.1–54.7) in Africa [19] and 48.3% (95% CI 22.3–66.1) in Asia [20]; while the efficacy of Rotarix in Malawi was 49.4% (95% CI 19.2–68.3) [21]. ROTAVAC, the only other rotavirus vaccine tested for efficacy in India, showed an efficacy of 53.6% (95% CI 35.0–66.9) [5]. Our results with BRV-PV in India are in line with these results. A recent study conducted in Niger with the same BRV-PV and under a design similar to that of our study, showed 66.7% efficacy against SRVGE and 78.8% against VSRVGE [22]. Differences in the population, the epidemiology of the disease, age at the time of SRVGE, and treatment practices may account for the observed difference in efficacy of BRV-PV between the two studies.

The reasons for the lower efficacy figures between developed and resource-limited countries have not been completely elucidated, however they may include high levels of transplacentally transmitted antibodies in the child gut, malnutrition, breast feeding, interfering gut flora, and co-infections, all common in resource-limited countries [23].

Of interest, the efficacy of RotaTeq against very severe, potentially life-threatening RVGE was 40.7% (95% CI 3.3–64.2) in Africa [19] and 70% (95% CI 31.8, 88.3) in Asia [20]. The corresponding figure for BRV-PV was 60.5% (95% CI 17.7, 81.0) in the period up to the primary analysis, when the children averaged 12.4 months of age and 52.9% for the entire 2-year follow-up period. The corresponding figures for ROTAVAC in India was 54.4% (95% CI 18.3–82.6) at the time of primary analysis [5] and 57.2% (95% CI 0.3 to 81.9) for the entire 2-year follow-up period [24]. At the other end of the severity spectrum, it is well known that even in developed countries RV vaccine efficacy is lower against less severe GE episodes; in our study, the efficacy of BRV-PV against RVGE of any severity at the time of the primary analysis (28% in the PP analysis, 35% in the ITT analysis) was in line with similar analysis for RotaTeq in Africa (30.5%) [19], ROTAVAC in India (36.4%) [5], Rotarix in Malawi (34.7%) [21], and BRV-PV in Niger (34.5%) [22]. The equivalent analysis for the entire follow-up period revealed an efficacy of 22.6% in the PP analysis and 24.2% in the ITT analysis, which is also in line with the results of observed with other vaccines.

Comparison of efficacy levels across trials of different rotavirus vaccines is challenging given the multiple variables involved (e.g., trial design, data collection, populations, disease epidemiology, burden of GE, associated interventions, etc.). An important factor to take into account in such comparisons is the type of surveillance applied to detect GE episodes. For example, in the RotaTeq studies in Africa where surveillance was passive (cases were detected when brought to medical facilities), a larger percentage of cases were of greater severity (61.6% of the cases were ≥11 with the Vesikari score) than those in this trial (43% of the cases scored ≥11). As noted above, vaccine efficacy estimates are typically higher for the cases of greater severity within the same study.

Another factor that affects comparisons of data across trials is the indiscriminate use of the Vesikari score whose application may differ widely across studies, and even across sites within a given study. There are seven scoring parameters included in the system: diarrhoea, vomiting, fever, dehydration, duration of diarrhoea, duration of vomiting, and treatment (rehydration and hospitalization) [11]. While evaluation of the first six parameters would appear to be objective, evaluation of the treatment parameter can vary greatly among health care providers. In our study, in which we were very cautious with respect to participant safety, we instituted oral rehydration therapy immediately at the onset of diarrhoea, a practice that may have influenced the course of the episode and thus lowered the potential score. Also, hospitalization and intravenous rehydration may have happened in some non-severe cases which might have inflated scores resulting in reduced vaccine efficacy for SRVGE. To avoid the potential biases introduced by these practices in the future, there is a need for more objective ways to assess disease severity in GE clinical trials, especially before interventions are started.

BRV-PV consists of G1, G2, G3, G4, and G9 serotypes, and our study found that G1, G2, G3, G9, and G12 were responsible for about 90% of the SRVGE in the study population, indicating that the vaccine can induce broad protection. This finding is important for potential use of the vaccine globally.

The BRV-PV was well tolerated when given together with DTP-HB-Hib and oral polio vaccines. The incidence of immediate solicited AEs, unsolicited AEs, SAEs, and deaths were similarly distributed in both the groups. The incidence of irritability was higher in the vaccine group, though this may be an incidental finding.

The only SAEs for which the vaccine was assessed by site investigators as causally related to study products were 12 cases of gastroenteritis that occurred within 7 days post-vaccination. Of these, 7 were in the BRV-PV group and 5 were in the placebo group. Incidentally, of these 12, only one tested positive while 11 tested negative for rotavirus antigen in stool by ELISA, thus making it unlikely that the illnesses were caused by the vaccine. Genotyping analysis of the single rotavirus positive case revealed it not to be the vaccine strain.

In some settings, post-marketing surveillance of the currently available rotavirus vaccines has detected a small increased risk of intussusception of about 1–2/100,000 infants vaccinated [25], [26], [27], [28]. In our study, no cases of intussusception occurred within 28 days of vaccinations. The incidence was also similar in BRV-PV and placebo groups. Though the study is not powered to discriminate for events of rare incidence, these findings are reassuring.

The rate of IgA seroresponses observed in this population (33%), while low, is similar to that of other rotavirus vaccine studies in India [29]. Responses to all three serotypes in the concomitantly administered oral poliovirus vaccine (OPV) were similar among placebo and vaccine recipients, demonstrating that BRV-PV did not interfere with OPV takes, a general concern that has been raised for other rotavirus vaccines, but which has not been substantiated for any [30], [31].

Transport and storage of vaccines at 2–8 °C has become an important challenge due to the introduction of newer vaccines [32], [33], [34], [35], especially for countries with insufficient cold-chain capacities [36], [37], [38], [39]. As a result, WHO has shown a preference for heat- and freeze-stable vaccines for supply to United Nations agencies [40]. BRV-PV is a highly heat-stable vaccine [41]. Based on the stability data, the recommendation for post-licensure use will be to store the vaccine below 25 °C. This will be very important in settings where power failures are common and where immunizations in remote villages are conducted in outreach programmes.

One potential ethical concern related to our study was that it was conducted as a placebo-controlled study. However, at the time of study launch, no licensed rotavirus vaccines that had undergone efficacy testing in India were available, and rotavirus vaccines were not yet a part of the routine immunization programme. Moreover, we maintained a high safety network in the study to ensure prompt and free treatment in case of any medical events. In addition, a WHO consultation held in 2013 confirmed this study design as ethically acceptable [42].

## 5 Conclusions

The BRV-PV developed in India was found efficacious and safe in Indian infants and can be a cost-effective and heat-stable option in the global strategy for diarrhoea prevention.
