# Supplementary material for: A randomized Phase III clinical trial to assess the efficacy of a bovine-human reassortant pentavalent rotavirus vaccine in Indian infants
Source: Vaccine. 2017 Oct 27;35(45):6228–37. doi: 10.1016/j.vaccine.2017.09.014 (PMC5651219; doi:10.1016/j.vaccine.2017.09.014)
Supplement: Supplementary data 1 [file mmc1.docx]

**Supplemental Table 1. Vaccine Efficacy (VE) by Severity Score - Intent to Treat Analysis**

| **Vesikari Score** | **Primary Analysis** | | | **Full 2-years follow-up** | | |
| --- | --- | --- | --- | --- | --- | --- |
|  | **n cases** | **Cum** | **Cum VE** | **n cases** | **Cum** | **Cum VE** |
| **>=16** | 39 | 39 | 61.3 | 99 | 99 | 52.9 |
| **15** | 30 | 69 | 53.9 | 71 | 170 | 46.4 |
| **14** | 26 | 95 | 45.1 | 62 | 232 | 40 |
| **13** | 34 | 129 | 43.6 | 83 | 315 | 39.7 |
| **12** | 22 | 151 | 40.4 | 76 | 396 | 37.7 |
| **11** | 24 | 175 | 41.9 | 86 | 482 | 38.8 |
| **1-10** | 232 | 407 | 35.3 | 729 | 1206 | 24.2 |

n= cases of rotavirus gastroenteritis (RVGEs); Cum= cumulative

**Supplemental Table 2. Efficacy of BRV-PV vaccine Against SRVGE by Genotype up to Two Years of Age – ITT population**

|  | **BRV-PV** | **Placebo** | **VE** | **VE 95% CI** |
| --- | --- | --- | --- | --- |
|  | **N=3749** | **N=3751** |  |  |
|  | **n(%)E** | |  |  |
| **All Genotypes** | 185 (4.93%), 228* | 291 (7.76%), 327 | 38.20% | (25.7, 48.6) |
| **G1** | 81 (2.16%), 82 | 129 (3.44%), 133 | 38.10% | (18.3, 53.1) |
| **G2** | 37 (0.99%), 39 | 60 (1.60%), 62 | 39.10% | (8.2, 59.5) |
| **G3** | 38 (1.01%), 38 | 42 (1.12%), 43 | 10.60% | (-38.7, 42.3) |
| **G9** | 54 (1.44%), 55 | 72 (1.92%), 74 | 25.90% | (-5.5, 47.9) |
| **G12** | 13 (0.35%), 13 | 18 (0.48%), 20 | 28.50% | (-45.9, 65.0) |

n=number of subjects in each category; E-Number of episodes of SRVGE for the number of subjects in each category

**Supplemental Table 3. Overview of Solicited Events: after dose 1, 2, 3 in the reactogenicity cohort**

| **Solicited AE Details** | **BRV-PV (N=500 )** | **Placebo (N=509 )** | **Total (N=1009)** | **P value (Fisher’s Exact test)** |
| --- | --- | --- | --- | --- |
| **n (%), E** | | | | |
| **At least one Solicited AE after dose 1** | 301 (60.2%), 589 | 316 (62.1%), 602 | 617 (61.1%), 1191 | 0.561 |
| **Number of Subjects with Solicited AE by Maximum Severity after dose 1** | | | | |
| Life-Threatening | 0 | 0 | 0 | --- |
| Severe | 2 (0.4%), 2 | 0 | 2 (0.2%), 2 | 0.245 |
| Moderate | 20 (4.0%), 20 | 30 (5.9%), 32 | 50 (5.0%), 52 | 0.192 |
| Mild | 279 (55.8%), 567 | 286 (56.2%), 570 | 565 (56.0%), 1137 | 0.949 |
| **At least one Solicited AE after dose 2** | 264 (53.7%), 438 | 238 (48.3%), 396 | 502 (51.0%), 834 | 0.098 |
| **Number of Subjects with Solicited AE by Maximum Severity after dose 2** | | | | |
| Life-Threatening | 0 | 0 | 0 | --- |
| Severe | 1 (0.2%), 1 | 1 (0.2%), 1 | 2 (0.2%), 2 | 1.000 |
| Moderate | 19 (3.9%), 19 | 15 (3.0%), 16 | 34 (3.5%), 35 | 0.492 |
| Mild | 244 (49.6%), 418 | 222 (45.0%), 379 | 466 (47.3%), 797 | 0.161 |
| **At least one Solicited AE after dose 3** | 197 (40.3%), 320 | 186 (38.8%), 283 | 383 (39.6%), 603 | 0.646 |
| **Number of Subjects with Solicited AE by Maximum Severity after dose 3** | | | | |
| Life-Threatening | 0 | 0 | 0 | --- |
| Severe | 1 (0.2%), 1 | 0 | 1 (0.1%), 1 | 1.000 |
| Moderate | 9 (1.8%), 10 | 12 (2.5%), 12 | 21 (2.2%), 22 | 0.515 |
| Mild | 187 (38.2%), 309 | 174 (36.3%), 271 | 361 (37.3%), 580 | 0.550 |

n= number of subjects with events; E= Number of all reported events including multiple occurrences of same event; %= Percentage is based on number of subjects in reactogenicity cohort for each group (N).

**Supplemental Table 4. Summary of Fatal Serious Adverse Events Post Dose 1 up to 2 Years of Age**

| **Fatal SAEs: Post Dose 1 up to 2 Years of Age (End of study)** | | | | |
| --- | --- | --- | --- | --- |
| **n (%), E** | | | | |
| **Serious Adverse Events** | **BRV-PV (N=3749)** | **Placebo (N=3751)** | **Total (N=7500)** | **P value (Fisher’s Exact test)** |
| Death | 3 (0.08%), 3 | 4 (0.11%), 4 | 7 (0.09%), 7 | 1.000 |
| Multi-organ failure | 0 | 1 (0.03%), 1 | 1 (0.01%), 1 | 1.000 |
| Sudden death | 0 | 1 (0.03%), 1 | 1 (0.01%), 1 | 1.000 |
| Sudden infant death syndrome | 1 (0.03%), 1 | 1 (0.03%), 1 | 2 (0.03%), 2 | 1.000 |
| Dengue fever | 1 (0.03%), 1 | 0 | 1 (0.01%), 1 | 0.500 |
| Encephalitis | 0 | 1 (0.03%), 1 | 1 (0.01%), 1 | 1.000 |
| Encephalitis viral | 0 | 1 (0.03%), 1 | 1 (0.01%), 1 | 1.000 |
| Meningitis tuberculous | 0 | 1 (0.03%), 1 | 1 (0.01%), 1 | 1.000 |
| Septic shock | 2 (0.05%), 2 | 0 | 2 (0.03%), 2 | 0.250 |
| Accidental poisoning | 0 | 1 (0.03%), 1 | 1 (0.01%), 1 | 1.000 |
| Drowning | 0 | 1 (0.03%), 1 | 1 (0.01%), 1 | 1.000 |
| Foreign body aspiration | 1 (0.03%), 1 | 0 | 1 (0.01%), 1 | 0.500 |
| Acute leukemia | 1 (0.03%), 1 | 0 | 1 (0.01%), 1 | 0.500 |
| Asphyxia | 1 (0.03%), 1 | 0 | 1 (0.01%), 1 | 0.500 |
| Aspiration | 1 (0.03%), 1 | 1 (0.03%), 1 | 2 (0.03%), 2 | 1.000 |
| Bronchiolitis | 1 (0.03%), 1 | 0 | 1 (0.01%), 1 | 0.500 |
| Bronchopneumonia | 1 (0.03%), 1 | 1 (0.03%), 1 | 2 (0.03%), 2 | 1.000 |
| Lobar pneumonia | 1 (0.03%), 1 | 0 | 1 (0.01%), 1 | 0.500 |
| Lower respiratory tract infection | 0 | 1 (0.03%), 1 | 1 (0.01%), 1 | 1.000 |
| Pneumonia | 1 (0.03%), 1 | 1 (0.03%), 1 | 2 (0.03%), 2 | 1.000 |
| Pneumonitis | 1 (0.03%), 1 | 0 | 1 (0.01%), 1 | 0.500 |

n= number of subjects with events; E= Number of all reported events; %= Percentage is based on number of subjects in the safety population for each group (N).
